# The renal nerves in chronic heart failure: efferent and afferent mechanisms

**DOI:** 10.3389/fphys.2015.00224

**Published:** 2015-08-07

**Authors:** Alicia M. Schiller, Peter R. Pellegrino, Irving H. Zucker

**Affiliations:** Cellular and Integrative Physiology, University of Nebraska Medical CenterOmaha, NE, USA

**Keywords:** heart failure, afferent pathways, efferent pathways, renal sympathetic nerves, renal denervation

## Abstract

The function of the renal nerves has been an area of scientific and medical interest for many years. The recent advent of a minimally invasive catheter-based method of renal denervation has renewed excitement in understanding the afferent and efferent actions of the renal nerves in multiple diseases. While hypertension has been the focus of much this work, less attention has been given to the role of the renal nerves in the development of chronic heart failure (CHF). Recent studies from our laboratory and those of others implicate an essential role for the renal nerves in the development and progression of CHF. Using a rabbit tachycardia model of CHF and surgical unilateral renal denervation, we provide evidence for both renal efferent and afferent mechanisms in the pathogenesis of CHF. Renal denervation prevented the decrease in renal blood flow observed in CHF while also preventing increases in Angiotensin-II receptor protein in the microvasculature of the renal cortex. Renal denervation in CHF also reduced physiological markers of autonomic dysfunction including an improvement in arterial baroreflex function, heart rate variability, and decreased resting cardiac sympathetic tone. Taken together, the renal sympathetic nerves are necessary in the pathogenesis of CHF via both efferent and afferent mechanisms. Additional investigation is warranted to fully understand the role of these nerves and their role as a therapeutic target in CHF.

## Introduction

Chronic heart failure (CHF) is a diverse clinical syndrome in which impairments of ventricular filling or emptying compromise the ability of the heart to match cardiac output to metabolic demand. This activates multiple maladaptive mechanisms such as inflammation, oxidative stress, the renin-angiotensin system (RAS), and the sympathetic nervous system (SNS), which over time contribute to disease progression (Felder et al., 2003; Tsutsui et al., 2011; Gullestad et al., 2012).

## Demographics of CHF

In the United States, approximately six million adults are living with CHF, a number that is expected to increase to over eight million in the next 15 years (Heidenreich et al., 2013). These patients suffer from dyspnea, fatigue, exercise intolerance, and edema, which degrade their quality of life and lead to frequent and costly hospitalizations. The American financial burden of CHF in 2012 was approximately $31 billion and is projected to balloon to nearly $70 billion by 2030 (Heidenreich et al., 2013). The prognosis of CHF is poor, with a 5-year mortality of approximately 50% (Roger et al., 2004), emphasizing the need for new therapeutic strategies.

## Cardiorenal interactions in CHF

Cardiac and renal dysfunction are inextricably intertwined in CHF, evidenced by the fact that over 80% of patients have renal insufficiency (McAlister et al., 2004). The hemodynamic disturbances of CHF lead to decreased renal blood flow with progressive proteinuria, diminished glomerular filtration rate, and renal fibrosis. Clinical studies have delineated an important relationship between markers of systemic venous congestion and renal function that is independent of other common prognostic markers (Mullens et al., 2008, 2009). Moreover, therapies that improve cardiac function, like cardiac resynchronization and left ventricular assist devices (LVAD), also improve renal function in CHF patients (Boerrigter et al., 2008; Sandner et al., 2009).

Several pieces of evidence implicate the kidney in the pathogenesis of CHF. Hypertension precedes 75% of incident CHF cases (Lloyd-Jones et al., 2002), and higher urinary albumin-to-creatinine ratios (Velagaleti et al., 2010) and hematocrit levels (Coglianese et al., 2012) are significant risk factors for the development of CHF. In established CHF, decreased glomerular filtration and increased renal sodium retention worsens the volume load on the already failing heart. In fact, renal dysfunction is a stronger predictor of mortality than New York Heart Association (NYHA) Functional Class and left ventricular ejection fraction (LVEF) in CHF patients (Hillege et al., 2000). The renal contribution to heart failure is further highlighted by a study of CHF patients on dialysis in whom renal transplantation improved LVEF from 32% pre-transplant to 52% 12 months post-transplant, normalizing cardiac function in nearly 70% of the cohort (Wali et al., 2005).

Despite the importance of hemodynamic dysregulation in CHF, the notion of heart failure as a purely hemodynamic disorder with purely hemodynamic cardiorenal interactions has been discredited (Bongartz et al., 2005; Bock and Gottlieb, 2010). Maladaptive mechanisms, including inflammation, oxidative stress, RAS activation, and sympatho-excitation, also drive morbidity and mortality in CHF patients.

## The sympathetic nervous system in chronic heart failure

Sympatho-excitation is a major component of the pathological relationship between the kidney and heart in CHF. The arterial baroreflex, chemoreflex, cardiac sympathetic afferent reflex, exercise pressor reflex, and cardiopulmonary reflexes modulate sympathetic outflow, and all of these reflexes are aberrant in CHF (Zucker et al., 1995). Sympatho-excitation activates the RAS and the immune system which increase sympatho-excitation in a deleterious feed forward fashion (Testa et al., 1996; Tsutsui et al., 2011; Sousa-Pinto et al., 2014). CHF is characterized by global sympatho-excitation, with cardiac and renal sympathetic outflows being particularly increased (Hasking et al., 1986), thus further implicating the SNS as an important mediator of deleterious cardiorenal interactions. Clinical evidence has demonstrated correlations between cardiac and renal sympathetic efferent activity and mortality in CHF (Brunner-La Rocca et al., 2001; Ogita, 2001; Petersson et al., 2005). A new minimally invasive therapeutic strategy targeting the renal sympathetic innervation has renewed interest in the role of the afferent and efferent renal nerves in health and disease.

## Rabbit tachycardia model as a model for human CHF

**Figure 1:**
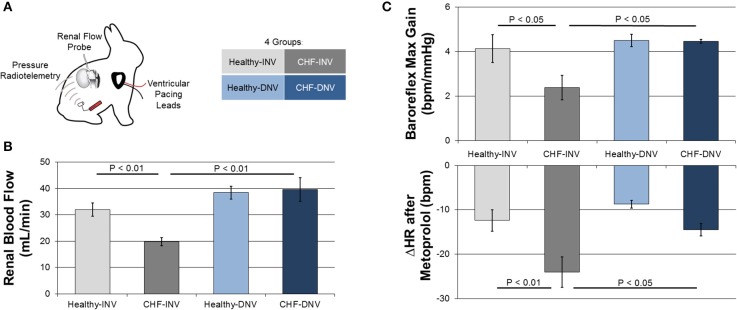
**Efferent and afferent signaling in the development of experimental CHF**. Rabbits were chronically instrumented with ventricular pacing leads, arterial pressure radiotelemeters, and renal flow probes, and underwent surgical denervation or a sham procedure **(A)**. Induction of CHF by ventricular pacing reduced blood flow in innervated (INV) rabbits but not in denervated (DNV) rabbits **(B)**. Modified from Clayton et al. (2011). Induction of CHF also reduced baroreflex gain and potentiated the heart rate response to metoprolol in INV but not DNV rabbits **(C)**. Modified from Schiller et al. (2013).

Recent studies from our laboratory investigated the role of the renal nerves in CHF in the rabbit rapid ventricular pacing model (Clayton et al., 2011; Schiller et al., 2013). The animals were chronically instrumented with ventricular pacing leads, arterial pressure radiotelemetries, and renal flow probes (Figure 1A). The kidney instrumented with a flow probe either underwent surgical denervation (DNV) or remained innervated (INV). CHF was induced over several weeks and validated by echocardiography. This model exhibits increases in renal sympathetic nerve activity (RSNA), plasma angiotensin II, and plasma norepinephrine (Mousa et al., 2008; Schiller et al., 2013), recapitulating the pathophysiology of human CHF. All studies were performed in the conscious state in rabbits that were well acclimated to the experimental procedures and environment.

## Efferent renal sympathetic nerve activity in CHF

The kidney receives rich sympathetic efferent innervation of many structures (Barajas et al., 1992). These renal sympathetic efferents mediate renal vasoconstriction, sodium reabsorption, and renin release.

Several studies indicate that the efferent renal nerves are essential to the renal hypoperfusion in CHF. Figure 1B shows the effect of renal denervation on renal blood flow (RBF) in our rabbit model of CHF. CHF significantly reduces RBF to INV but not DNV kidneys by increasing renal vascular resistance. These data support the necessity of the renal nerves in the development of renal hypoperfusion in CHF. Taken with the observation that acute renal denervation of anesthetized rats significantly increases RBF in CHF but not control rats (Kon et al., 1985; DiBona and Sawin, 2004), it seems the renal nerves exert a tonic vasoconstrictive action in CHF. Importantly, Kon et al. also found that acute renal denervation increases glomerular filtration rate while reducing glomerular capillary pressure in CHF, providing evidence that the elevated efferent RSNA in CHF impairs renal function.

Several studies have examined the contribution of the renal nerves to volume dysregulation in CHF. Renal denervation does not affect sodium excretion or balance in CHF animals unless they are challenged, for example by sodium restriction (DiBona and Sawin, 1991) or volume or salt loading (DiBona et al., 1988; Villarreal et al., 1994; Souza et al., 2004). Regardless of the challenge, denervated animals with CHF excrete more sodium than their innervated counterparts. A recent rodent study suggests that this is because the increased efferent RSNA in CHF increases Na^+^-K^+^-2Cl^−^ co-transporter expression in the thick ascending limb (Torp et al., 2012).

One study has shown that renal denervation attenuates but does not normalize the increased plasma renin activity in experimental CHF (Witty et al., 1972), indicating that the renal nerves are partly responsible for the maladaptive activation of the systemic RAS. In summary, efferent RSNA may contribute to the renal hypoperfusion, volume dysregulation, and RAS activation in CHF.

## Afferent renal sympathetic nerve activity in CHF

The kidney is heavily innervated with sensory nerves that transmit information from the renal chemo- and mechano-sensitive receptors that monitor parameters including composition of the interstitial fluid and hydrostatic pressure changes (Nijima, 1971; Uchida et al., 1971; Recordati et al., 1981). Activation of the renal afferent nerves modulates hypothalamic activity, pain sensation, and sympathetic outflow to multiple organs, including the heart and kidney (Stella and Zanchetti, 1991; Xu et al., 2015). While much of the attention on therapeutic renal denervation has focused on the role of efferent renal nerves in cardiovascular disease, renal denervation was first used clinically to eliminate renal afferent signaling to alleviate kidney pain (Oldham, 1950). Evidence of the importance of renal afferent signaling in hypertensive patients has brought more focus to this limb of the renal nerves in other cardiovascular diseases (Hering et al., 2013).

Given the ability of the afferent renal nerves to modulate central reflexes and sympathetic outflow, we investigated the effects of renal denervation on common markers of autonomic dysfunction in the rabbit pacing model of CHF (Schiller et al., 2013). Rabbits were administered an intravenous bolus of metoprolol, which results in a decrease in heart rate that is proportional to the resting cardiac sympathetic tone. The response to metoprolol was greater for CHF-INV rabbits than healthy rabbits, consistent with elevated cardiac sympathetic tone in CHF, while CHF-DNV rabbits had a significantly attenuated metoprolol response compared to CHF-INV rabbits (Figure 1C). This indicates that the renal nerves play a role in the development of the cardiac sympatho-excitation in CHF, most likely due to increased renal afferent feedback.

In the same rabbits, cardiac baroreflex control was assessed by infusion of vasoactive drugs. CHF-INV rabbits exhibited decreased baroreflex gain, which was prevented in CHF-DNV rabbits (Figure 1C). These data indicate that renal afferent signaling is important in the development of baroreflex dysfunction in CHF. Additionally, CHF-DNV rabbits had improved heart rate variability compared to CHF-INV rabbits, further implicating the renal nerves in the development of cardiac autonomic dysfunction in CHF. Cardiac sympathetic tone, and baroreflex sensitivity, and heart rate variability are prognostic for risk of sudden cardiac death in CHF. Another study in anesthetized pigs found that renal denervation decreased ventricular arrhythmias in response to acute ventricular ischemia (Linz et al., 2013), bolstering the idea that renal afferent signaling may be pro-arrhythmogenic in CHF. Cumulatively, these data suggest that the renal sympathetic afferents play a role in the development of autonomic dysfunction and possibly sudden cardiac death in CHF.

A recent study employed catheter-based renal denervation after the development of CHF in sheep (Booth et al., 2015b). Despite convincing evidence of complete functional afferent and efferent renal denervation as well as a drop in blood pressure, renal denervation did not decrease cardiac sympathetic tone or improve cardiac baroreflex gain in CHF sheep. The findings of this study contrast from our own data, but many possible explanations for these differences exist. First, the experiments are performed the day after denervation whereas ours were performed several weeks after denervation, and the short-term effects of nerve ablation may be markedly different from the long-term effects as time is required for neural circuit reorganization and renal and systemic molecular changes. Second, the ovine CHF model does not exhibit increased RSNA, and renal afferent signaling may not be important for autonomic dysfunction in this model. Along these lines, model-specific contributions of the renal afferents to hypertension has been recently demonstrated (Foss et al., 2015). Finally, because renal denervation was performed after induction of CHF, this study raises the very real possibility that renal afferent nerves are necessary for the development of autonomic dysfunction in CHF but do not maintain the autonomic dysfunction in established CHF. The reconciliation of this study with our own has important bearing on the viability of the renal nerves as a therapeutic target in CHF.

## Renal sympathetic nerves and neurohumoral activation in CHF

Evidence from our studies also indicates that the renal nerves play an important role in the neurohumoral activation of CHF mediated by both efferent and afferent mechanisms. CHF-INV rabbits had increased plasma norepinephrine which was normalized in CHF-DNV rabbits (Schiller et al., 2013). Furthermore, our laboratory has shown that renal denervation prevents deleterious molecular changes in the renal cortical microvasculature, which may be influenced by both local and circulating factors. Specifically, CHF-INV rabbits exhibited increased expression of the vasoconstrictive, profibrotic Angiotensin-II Type 1 Receptor (AT_1_R) compared to healthy rabbits (Clayton et al., 2011) while CHF-DNV rabbits did not show an increase in AT_1_R protein. Of note, this model, like human CHF, exhibits increases in circulating Angiotensin-II, which can, in turn, upregulate the deleterious AT_1_R in a feed-forward manner (Zucker et al., 2001). Whether these changes are a local phenomenon or if similar effects can be seen throughout the vasculature of denervated animals awaits further investigation.

## Renal sympathetic nerves and cardiac function in CHF

In our rapid ventricular pacing model of CHF, the degree of heart failure is verified by echocardiography, and rabbits are defined to be in CHF after their LVEF falls below a threshold. Thus, cardiac function is a controlled parameter, and our studies are unable to answer questions about the role of the renal nerves in the development of cardiac dysfunction. However, in a rat myocardial infarction CHF model, chronic renal denervation decreased left ventricular end-diastolic diameter and increased left ventricular fractional shortening (Nozawa et al., 2002), indicating that the renal nerves play a role in the development of cardiac dysfunction in this model suggesting that they are important in the feed-forward pathophysiology of CHF.

## The renal nerves as a therapeutic target in CHF

Taken together, the above studies implicate both the afferent and efferent renal nerves in the development of the pathophysiology of CHF. Despite importance of the renal nerves in the development of CHF, several open questions remain about their potential as a therapeutic target. Specifically, the efficacy and longevity of catheter-based renal denervation and the role of the renal nerves in established CHF are points of further discussion.

## Catheter-based renal denervation

In most pre-clinical studies, renal denervation is performed surgically by stripping the neural tissue from the renal artery (Clayton et al., 2011; Schiller et al., 2013). The result is essentially complete denervation, evidenced by nearly 100% reductions in renal cortical norepinephrine levels in the rabbit, dog, and rat (Lohmeier et al., 2007; Clayton et al., 2011; Schiller et al., 2013; Linz et al., 2015). This contrasts from the partial denervation achieved by bilateral radiofrequency ablation with a catheter which is performed in patients. The only clinical study that has directly addressed the efficacy of catheter-based renal denervation was performed on 10 patients who underwent renal denervation with the Medtronic Symplicity™ catheter in whom renal norepinephrine spillover was assessed before and 15–30 days after denervation (Krum et al., 2009). After denervation, renal norepinephrine spillover was reduced by an average of 47%, but this response was highly variable between subjects. Much better results have been achieved in both sheep and pigs (Booth et al., 2015b), but the fact that the renal nerves do not form a network around the main renal artery in man may render such complete denervation impossible (Oldham, 1950). The partial efficacy of catheter-based techniques in patients contrasts from surgical denervation, which consistently and completely eliminates renal norepinephrine in all species. As it stands, it appears that partial denervation is the best that can be achieved by catheter-based renal ablation in humans, but this may be sufficient for a therapeutic benefit, as evidenced by our studies of unilateral denervation.

In addition to concerns about the completeness of catheter-based renal denervation, the duration of denervation in patients remains unknown. The longevity of afferent and efferent denervation after both surgical and catheter-based denervation of animals is only a few months (Mulder et al., 2013; Booth et al., 2015a). In renal transplant patients, histochemical studies have shown abundant allograft innervation at 8 months post-transplant (Gazdar and Dammin, 1970), but this innervation may not be functional even years after transplantation (Hansen et al., 1994). The time course of both molecular and functional renal reinnervation after catheter-based denervation remains to be investigated and will certainly impact the therapeutic potential of this technique.

## The renal nerves in established CHF

A very important point that our studies were not designed to address is whether or not the renal nerves remain a therapeutic target in established CHF. We performed surgical renal denervation prior to ventricular pacing, and thus the findings in our studies represent the cumulative contribution of the renal nerves through the development of CHF. This does not necessarily mean that the renal nerves actively drive these same processes in established CHF, a distinction which is emphasized by studies in other disease models in which renal denervation is preventative but not therapeutic (Stella and Zanchetti, 1991; Kim and Padanilam, 2013) and has been suggested by the aforementioned short-term study in sheep with CHF (Booth et al., 2015b). Whether or not renal denervation performed after the development of CHF can slow or reverse the renal hypoperfusion, autonomic imbalance, and neurohumoral activation in CHF remains an open question.

## Clinical studies of renal denervation in CHF

The excitement surrounding the Medtronic Symplicity renal nerve ablation catheter in the wake of promising Phase 1 and 2 clinical trials in hypertension gave way to despair after the Phase 3 SYMPLICITY HTN-3 failed to achieve its efficacy endpoints (Bhatt et al., 2014). The reasons for the disparities between the earlier trials and SYMPLICITY HTN-3 have been discussed at length by many other authors (Esler, 2014; Kandzari et al., 2015), and include completeness of denervation, patient selection, the placebo effect, and in-trial medication regimen changes (Bhatt and Bakris, 2014). Recently, DENERHTN, which was not sham-controlled but was carried out at select hypertension centers by experienced interventionalists in patients on a carefully controlled medication regimen, showed that the addition of renal denervation to a standard medication regimen significantly decreased 24-h ambulatory systolic blood pressure (Azizi et al., 2015). Despite questions about its efficacy, catheter-based denervation is generally safe. HTN-3 showed that denervation was no worse than renal angiography in terms of renovascular complications, renal function, all-cause mortality, and cardiovascular events, while two patients in DENERHTN experienced lumbar pain and one developed a groin hematoma.

Presently, ClinicalTrials.gov lists 18 studies for renal denervation in CHF. Only one, a small pilot safety study in seven CHF patients, has been completed (Davies et al., 2013). These patients did not have hypotensive complications or deterioration of renal function after renal denervation with the Medtronic Symplicity™ catheter. All patients felt better after denervation and improved their 6-min walk distance despite no change in echocardiographic parameters. Many of the other studies are aimed at particular CHF subgroups, including heart failure with preserved ejection fraction, CHF with renal impairment, and CHF secondary to Chagas disease. Also notable is that some of these trials were withdrawn or terminated following the efficacy shortcomings of SYMPLICITY HTN-3.

## Conclusion

**Figure 2:**
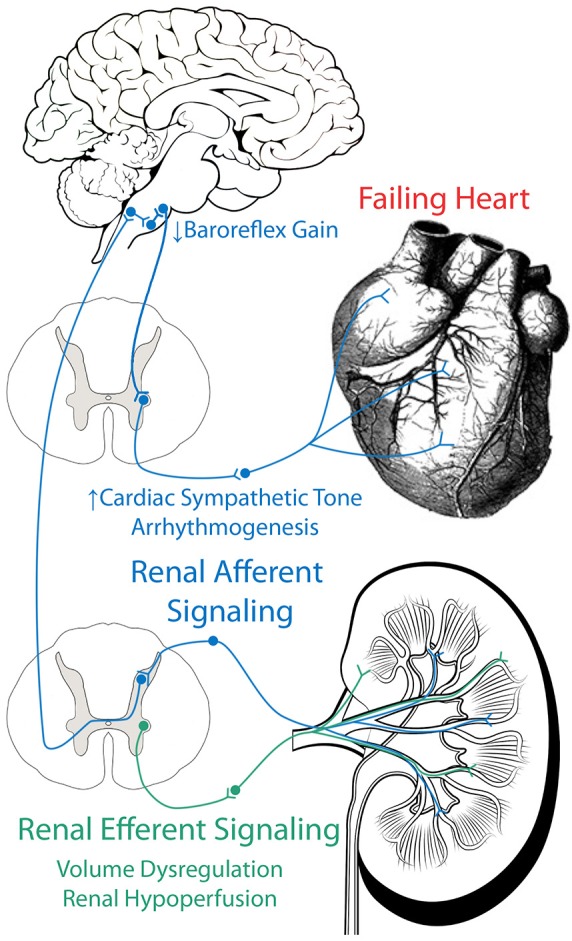
**Role of efferent and afferent renal nerves in CHF**. Efferent renal signaling decreases renal blood flow and contributes to volume dysregulation. Afferent renal signaling contributes to cardiac sympatho-excitation, baroreflex dysfunction, and arrhythmogenesis.

We have reviewed evidence which implicates both the efferent and afferent renal sympathetic nerves in the development several of the pathophysiological hallmarks of CHF, including renal hypoperfusion, volume dysregulation, cardiac sympatho-excitation, baroreflex dysfunction, arrhythmogenesis, and neurohumoral activation (Figure 2). More research, both basic and clinical, is necessary to determine if and exactly how renal denervation can be employed as an effective therapeutic strategy for CHF patients.

## Funding

This work was supported by a National Institutes of Health National Heart, Lung, and Blood Institute (NIH NHLBI) Grant P01 HL62222. AS was supported by an American Heart Association pre-doctoral fellowship (13PRE14700045); PP was supported by a NIH NHLBI F30 pre-doctoral fellowship (1F30HL118974-01A1).

### Conflict of interest statement

The authors declare that the research was conducted in the absence of any commercial or financial relationships that could be construed as a potential conflict of interest.
